# Molecular epidemiological and pharmaceutical studies of methicillin-resistant *Staphylococcus aureus* isolated at hospitals in Kure City, Japan

**DOI:** 10.1099/acmi.0.000319

**Published:** 2022-02-25

**Authors:** Ryuto Maeda, Hidetomo Kobayashi, Mami Higashidani, Tetsuaki Matsuhisa, Akihiro Sawa, Katsushi Miyake, Yoshitaka Tayama, Kouji Kimura, Hiroyuki Itoh, Taichi Okano, Soshi Seike, Hiroyasu Yamanaka

**Affiliations:** ^1^​ Research Center for Pharmaceutical Health Care and Sciences, Faculty of Pharmaceutical Sciences, Hiroshima International University, Hiro-Koshingai, Kure, Hiroshima 737-0112, Japan; ^2^​ National Hospital Organization, Kure Medical Center and Chugoku Cancer Center, Aoyama-cho, Kure, Hiroshima 737-0023, Japan; ^3^​ Laboratory of Molecular Microbiological Science, Faculty of Pharmaceutical Sciences, Hiroshima International University, Hiro-Koshingai, Kure, Hiroshima 737-0112, Japan; ^4^​ Saiseikai Kure Hospital, Sanjo, Kure, Hiroshima 737-0821, Japan

**Keywords:** *Staphylococcus aureus*, MRSA, genome typing, pulse-field gel electrophoresis, epidemiology, phylogeny, SCC*mec*, antibacterial agents, antibiotic resistance, AUD/DOT value

## Abstract

**Introduction:**

Methicillin-resistant *Staphylococcus aureus* (MRSA) is one of the major pathogens of nosocomial infections throughout the world. In the medical field, it is extremely important to this pathogen’s trends when considering infection control.

**Hypothesis/Gap Statement:**

We hypothesized that clarifying the characteristics of clinically isolated MRSA would contribute to infection control and proper use of antimicrobial agents against MRSA.

**Aim:**

The purpose of this study is to elucidate the genetic and biological characteristics of the MRSA isolates found at our hospital and to reveal changes in the spread of this pathogen in the local area where we live.

**Methodology:**

Pulse-field gel electrophoresis (PFGE) and polymerase chain reaction were used for the genetic analyses of MRSA isolates. Toxin production by each isolate was examined using toxin-specific detection systems.

**Results:**

During the 3 years from 2017 through 2019, over 1000 MRSA strains were isolated at our hospital. Genomic analysis of 237 of these clinical isolates by PFGE revealed 12 PFGE types (types A to L), each consisting of five or more MRSA clinical strains with over 80% genetic similarity. Examination of the SCC*mec* genotypes found that 219 of 237 isolated MRSA strains (approximately 92%) were SCC*mec* genotype II or IV and that only four of the isolates carried the Panton−Valentine leukocidin (PVL) gene. Examination of the toxin production of the isolates using staphylococcal enterotoxin detection kits found that most isolates carrying the SCC*mec* genotype II produced enterotoxin B and/or C, and that most isolates carrying the SCC*mec* genotype IV produced enterotoxin A.

**Conclusion:**

The present results revealed that MRSA isolates with common properties were isolated at certain rates throughout the 3 year study period, suggesting that relatively specific MRSA clones may have settled in the local area around our hospital. We also examine the relationship between antimicrobial usage over time and changes in MRSA isolation rates.

## Introduction

Methicillin-resistant *Staphylococcus aureus* (MRSA) is still globally regarded as the main causative agent of nosocomial infections. According to data from the ‘Annual Open Report 2019’ published by the Japan Nosocomial Infection Surveillance (JANIS) project of the Ministry of Health, Labour and Welfare, Japan, MRSA remains the most frequently isolated drug-resistant bacterial strain in Japanese hospitals, accounting for 93.35% (17134 inpatients) of the total of 18354 inpatients that had new infections of all drug-resistant bacterial strains. Thus, the clinical problems related to MRSA are far from being resolved even in Japan, and close attention to it will still need to be paid in the future.

MRSA first appeared in the early 1960s, and several epidemic health care-associated MRSA (HA-MRSA) clones have emerged since the 1970s. From the 1990s on, however, several community-associated MRSA (CA-MRSA) clones have spread worldwide [1, 2]. Popovich *et al*. reported that the USA300 MRSA strain, the most common CA-MRSA strain in the United States, was displacing traditional hospital MRSA strains and causing new health and hygiene problems among some races [3]. In addition, due to the clinical use of various antibacterial agents, new MRSA clones have been also emerging [4, 5]. MRSA is spread all over the world [2], and the strain map of MRSA isolates from Asia and the Pacific is especially diverse [6–9]. Since the genetic evolution of MRSA is likely to continue, molecular epidemiological surveillance of this pathogen will become increasingly important.

Resistance to β-lactam antibiotics is caused by the *mec*A gene, which is positioned at a mobile genetic element, the Staphylococcal Cassette Chromosome *mec* (SCC*mec*). Five SCC*mec* types, SCC*mec* types I to V, have been known as typical SCC*mec* genotypes [10, 11], and several of their variants have been further reported [12–19]. Staphylococci other than MRSA are also known to have SCC*mec*, and there is concern that they may be new suppliers of drug resistance gene transfer [20, 21]. It is therefore important to always pay attention to the genetic features of MRSA isolated in the medical field.

Pulse-field gel electrophoresis (PFGE) is a widely used method of analysing the genomic properties of MRSA. In combination with this molecular typing method, SCC*mec* typing, multilocus sequence typing (MLST), and typing of the variable tandem repeat region of protein A (spa typing) are widely used to analyse the characteristics of MRSA [22]. MRSA characteristics are also analysed by Panton−Valentine leukocidin (PVL) gene detection, staphylococcal coagulase gene (*coa*) typing, and assays for the production of various toxins such as exfoliative toxins, enterotoxins, and toxic shock syndrome toxins [2]. We therefore applied these methods to analyse clinically isolated MRSA strains.

In this study, we first performed molecular epidemiological analysis of MRSA clinical strains isolated from 2017 through 2019 at the National Hospital Organization Kure Medical Centre (NHOKMC) in Kure city, Hiroshima prefecture, Japan. NHOKMC has 35 clinical departments and 700 beds. We further examined the possession of the *pvl* gene in the MRSA isolates by PCR and the production of several enterotoxins, toxic syndrome toxin-1 and exfoliative toxin using their respective toxin detection kits. In addition, we also investigated the relationship between the incidence of MRSA infections and the usage of antibacterial agents in our hospital, in parallel with the above basic research. Finally, we attempted to comprehensively discuss their results. Although this research is a limited study performed at one medical institution, it is a very important effort to track changes in the characteristics of MRSA isolated in medical facilities that are visited by many patients, including outpatients, and to understand MRSA trends. From this point of view, we believe that continuing such studies will provide valuable information with which to control MRSA infection.

## Methods

### Bacterial isolates examined in this study

The representative MRSA clinical isolates classified into 12 PFGE types (types A to L) in this molecular epidemiological study are listed in Table 1 along with the origins of the specimens. These isolates are among the MRSA clinical strains isolated at NHOKMC from 2017 through 2019. The sensitivity of each isolate to various antibacterial agents assessed from the MIC (minimum inhibitory concentration) values is also specified in Table S1 (available in the online version of this article), sensitive; I, intermediate; R, resistant). When the MIC value reached the value shown in the margin of Table 1, it was judged to be R (resistant to each antibacterial agent). MRSA was determined to have an oxacillin (MPIPC) MIC value of 4 µg ml^−1^ or higher based on the NCCLS (National Committee for Clinical Laboratory Standards) criteria. The MRSA isolation rate (%) was calculated as the rate of the number of MRSA isolates to the total number of *S. aureus* isolated from the patients at our hospital.

**Table 1.** Drug resistance findings of MRSA clinical strains and their origins of their samples

PFGE Type	Strain number	Collection date (Year/Month/Day)	Origin of the sample	Age	Gender	Resistance to antibacterial agents [R, resistant (filled with gray); S, sensitive; I, intermediate]																									
						ABPC	MPIPC	AZM	PCG	CEZ	CTM	EM/ CLDM	DAP	CFDN	CFPM	CMZ	FMOX	IPM	MEPM	ABPC/SBT	CPZ/ SBT	PIPC/ TAZ	ABK	GM	CLDM	MINO	TEIC	VCM	CPFX	LVFX	FOM
	KI-101	2018/ 3/26	Pharyngeal mucus	71	M	R	R	R	R	R	R	R	S	R	R	R	R	R	R	R	R	R	S	R	R	R	S	S	R	R	S
	KI-203	2019 /7/1	Sputum	81	M	R	R	R	R	R	R	R	S	R	R	R	R	R	R	R	R	R	S	R	R	R	S	S	R	R	R
A	KI-230	2019/ 11/5	Sputum	79	F	R	R	R	R	R	R	R	S	R	R	R	R	R	R	R	R	R	S	R	R	R	S	S	R	R	S
	KI-234	2019/ 11/15	Intratracheal sputum	59	M	R	R	R	R	R	R	R	S	R	R	R	R	R	R	R	R	R	S	R	R	R	S	S	R	R	R
	KI-239	2019/ 12/16	Sputum	90	M	R	R	R	R	R	R	R	S	R	R	R	R	R	R	R	R	R	S	R	R	R	S	S	R	R	R
	KI-127	2018/ 7/12	Natural urination	85	M	R	R	I	I	R	R	R	S	R	R	R	R	R	R	R	R	R	S	S	R	I	S	S	R	R	S
	KI-137	2018/ 9/4	Obstructive pus	77	F	R	R	R	R	R	R	R	S	R	R	R	R	R	R	R	R	R	S	S	R	R	S	S	R	R	R
	KI-150	2018 /11/13	Obstructive pus	47	M	R	R	R	R	R	R	R	S	R	R	R	R	R	R	R	R	R	S	S	R	R	S	S	R	R	I
B	KI-151	2018/ 11/19	Indwelling catheter urine	85	M	R	R	R	R	R	R	R	S	R	R	R	R	R	R	R	R	R	S	R	R	S	S	S	R	R	S
	KI-153	2018/ 11/27	Ear secretions	64	M	R	R	R	R	R	R	R	S	R	R	R	R	R	R	R	R	R	S	R	R	R	S	S	R	R	S
	KI-155	2018/ 12/4	Ear secretions	76	F	R	R	R	R	R	R	R	S	R	R	R	R	R	R	R	R	R	S	R	R	R	S	S	R	R	R
	KI-007	2017/ 1/11	Sputum	81	M	R	R	R	R	R	R	R	S	R	R	R	R	R	R	R	R	R	S	S	R	S	S	S	R	I	S
	KI-012	2017/ 2/10	Pressure ulcer	80	F	R	R	R	R	R	R	R	S	R	R	R	R	R	R	R	R	R	S	S	R	S	S	S	R	R	S
	KI-016	2017/ 3/1	Sputum	87	M	R	R	R	R	R	R	R	S	R	R	R	R	R	R	R	R	R	S	R	R	I	S	S	R	R	R
	KI-021	2017/ 4/3	Abdominal drain tip	74	M	R	R	R	R	R	R	R	S	R	R	R	R	R	R	R	R	R	S	R	R	R	S	S	R	R	S
C	KI-028	2017/ 5/2	Indwelling catheter urine	42	F	R	R	R	R	R	R	R	S	R	R	R	R	R	R	R	R	R	S	R	R	R	S	S	R	R	R
	KI-043	2017 /7/3	Skin	58	M	R	R	R	R	R	R	R	S	R	R	R	R	R	R	R	R	R	S	R	R	R	S	S	R	R	S
	KI-045	2017/ 7/4	Sputum	74	F	R	R	R	R	R	R	R	S	R	R	R	R	R	R	R	R	R	I	R	R	I	S	S	R	R	S
	KI-050	2017/ 8/8	Sputum	76	M	R	R	R	R	R	R	R	S	R	R	R	R	R	R	R	R	R	S	R	R	R	S	S	R	R	S
	KI-058	2017/ 9/6	Feces	60	M	R	R	R	R	R	R	R	S	R	R	R	R	R	R	R	R	R	S	S	R	R	S	S	R	R	I
	KI-063	2017/ 10/6	Indwelling catheter urine	92	F	R	R	R	R	R	R	R	S	R	R	R	R	R	R	R	R	R	S	R	R	R	S	S	R	R	R
	KI-132	2018/ 8/16	Open pus	63	M	R	R	I	R	R	R	R	S	R	R	R	R	R	R	R	R	R	S	S	R	S	S	S	R	R	S
	KI-138	2018 /9/5	Sputum	84	M	R	R	R	R	R	R	R	S	R	R	R	R	R	R	R	R	R	S	S	R	S	S	S	R	R	S
D	KI-201	2019/ 6/15	Venous blood	73	M	R	R	R	R	R	R	R	S	R	R	R	R	R	R	R	R	R	S	S	R	S	S	S	R	R	S
	KI-220	2019/ 9/10	Sputum	86	F	R	R	R	R	R	R	R	S	R	R	R	R	R	R	R	R	R	S	S	R	S	S	S	R	R	S
	KI-224	2019/ 10/11	Sputum	80	M	R	R	R	R	R	R	R	S	R	R	R	R	R	R	R	R	R	S	S	S	S	S	S	R	R	S
	KI-236	2019/ 12/6	Intratracheal sputum	89	M	R	R	R	R	R	R	R	S	R	R	R	R	R	R	R	R	R	S	S	R	S	S	S	R	R	S
	KI-126	2018/ 7/12	Open pus	65	M	R	R	R	R	R	R	R	S	R	R	R	R	R	R	R	R	R	S	S	R	S	S	S	R	R	S
	KI-142	2018/ 9/22	Venous blood	74	F	R	R	R	R	I	R	R	S	R	R	R	R	R	R	R	R	R	S	S	R	S	S	S	R	R	S
E	KI-143	2018/ 10/1	Indwelling catheter urine	74	M	R	R	I	R	R	R	I	S	R	R	R	R	R	R	R	R	R	S	S	S	S	S	S	R	R	S
	KI-145	2018/ 10/3	Wound	75	M	R	R	R	R	R	R	R	S	R	R	R	R	R	R	R	R	R	S	S	R	S	S	S	R	R	S
	KI-146	2018/ 10/3	Urine catheter	59	M	R	R	R	R	I	R	R	S	R	R	R	R	R	R	R	R	R	S	S	R	S	S	S	R	R	S
	KI-152	2018/ 11/19	Indwelling catheter urine	88	M	R	R	S	R	R	R	I	S	R	R	R	R	R	R	R	R	R	S	S	S	S	S	S	R	R	S
	KI-019	2017/ 3/10	Venus blood	83	F	R	R	S	R	I	R	I	S	R	R	R	R	R	R	R	R	R	S	S	S	S	S	S	R	R	S
	KI-020	2017/ 4/3	Obstructive pus	32	F	R	R	R	R	R	R	R	S	R	R	R	R	R	R	R	R	R	S	S	R	S	S	S	R	R	S
F	KI-031	2017/ 5/9	Open pus	71	F	R	R	R	R	R	R	R	S	R	R	R	R	R	R	R	R	R	S	S	R	S	S	S	S	S	R
	KI-083	2018/ 1/15	Skin	87	F	R	R	R	R	I	R	I	S	R	R	R	R	R	R	R	R	R	S	S	S	S	S	S	R	R	S
	KI-087	2018/ 1/23	Venus blood	80	F	R	R	I	R	R	R	I	S	R	R	R	R	R	R	R	R	R	S	S	S	S	S	S	R	R	S
	KI-044	2017/ 7/5	Open pus	81	F	R	R	S	R	R	R	I	S	R	R	R	R	R	R	R	R	R	S	S	S	S	S	S	R	R	S
	KI-053	2017/ 8/14	Intratracheal sputum	93	M	R	R	R	R	R	R	R	S	R	R	R	R	R	R	R	R	R	S	S	R	S	R	S	R	R	S
	KI-054	2017/ 9/4	Sputum	71	M	R	R	R	R	R	R	R	S	R	R	R	R	R	R	R	R	R	S	S	R	S	R	S	R	R	S
G	KI-055	2017/ 9/4	Venus blood	71	M	R	R	R	R	R	R	R	S	R	R	R	R	R	R	R	R	R	S	S	R	S	R	S	R	R	S
	KI-056	2017/ 9/5	Sputum	83	F	R	R	R	R	R	R	R	S	R	R	R	R	R	R	R	R	R	S	S	R	S	R	S	R	R	S
	KI-067	2017/ 10/13	Feces	59	F	R	R	R	R	R	R	R	S	R	R	R	R	R	R	R	R	R	S	S	R	S	R	S	R	R	S
	KI-079	2017/ 12/6	Nasal cavity	0	F	R	R	R	R	R	R	R	S	R	R	R	R	R	R	R	R	R	S	S	R	S	R	S	R	R	S
	KI-084	2018/ 1/17	Sputum	82	F	R	R	R	R	R	R	R	S	R	R	R	R	R	R	R	R	R	S	S	R	I	S	S	R	I	S
	KI-115	2018/ 5/14	Intratracheal sputum	80	F	R	R	R	R	R	R	R	S	R	R	R	R	R	R	R	R	R	S	R	R	S	S	S	R	R	S
	KI-139	2018/ 9/10	Urine catheter	88	M	R	R	R	R	R	R	R	S	R	R	R	R	R	R	R	R	R	S	R	R	S	S	S	R	R	S
	KI-210	2019/ 8/2	Vaginal discharge	39	F	R	R	R	R	R	R	R	S	R	R	R	R	R	R	R	R	R	S	S	R	S	S	S	R	R	S
H	KI-213	2019/ 8/7	Natural urination	70	F	R	R	R	R	R	R	R	S	R	R	R	R	R	R	R	R	R	S	R	R	S	S	S	R	R	S
	KI-227	2019/ 10/21	Bronchial sputum	74	M	R	R	R	R	R	R	R	S	R	R	R	R	R	R	R	R	R	S	R	R	S	S	S	R	R	S
	KI-228	2019/ 10/23	Bronchial sputum	71	M	R	R	R	R	R	R	R	S	R	R	R	R	R	R	R	R	R	S	R	R	S	S	S	R	R	S
	KI-235	2019/ 12/2	Vaginal discharge	26	F	R	R	R	R	R	R	R	S	R	R	R	R	R	R	R	R	R	S	R	R	S	S	S	R	R	S
	KI-110	2018/ 5/7	Ear secretions	82	F	R	R	S	R	I	R	I	S	R	R	R	R	R	R	R	R	R	S	R	S	S	S	S	R	R	S
	KI-178	2019/ 3/6	Sputum	73	F	R	R	S	R	R	R	I	S	R	R	R	R	R	R	R	R	R	S	R	S	S	S	S	R	I	S
I	KI-182	2019/ 4/2	Sputum	83	M	R	R	S	R	I	R	I	S	R	R	R	R	R	R	R	R	R	S	S	S	S	S	S	R	R	S
	KI-187	2019/ 4/10	Venous blood	88	M	R	R	R	R	R	R	R	S	R	R	R	R	R	R	R	R	R	S	I	R	S	S	S	R	R	S
	KI-192	2019/ 5/13	Sputum	88	F	R	R	S	R	R	R	I	S	R	R	R	R	R	R	R	R	R	S	R	S	S	S	S	R	R	S
	KI-161	2019/ 1/4	Open pus	70	F	R	R	S	R	R	R	I	S	R	R	R	R	R	R	R	R	R	S	R	S	S	S	S	S	S	S
	KI-173	2019/ 2/25	Sputum	48	F	R	R	S	R	R	R	I	S	R	R	R	R	R	R	R	R	R	S	R	S	S	S	S	S	S	S
J	KI-181	2019/ 3/8	Sputum	63	M	R	R	S	R	R	R	I	S	R	R	R	R	R	R	R	R	R	S	S	S	S	I	S	S	S	S
	KI-186	2019/ 4/8	Intratracheal sputum	75	M	R	R	R	R	R	R	R	S	R	R	R	R	R	R	R	R	R	S	R	R	R	S	S	R	R	S
	KI-199	2019/ 6/12	Ear secretions	79	M	R	R	R	R	R	R	R	S	R	R	R	R	R	R	R	R	R	S	R	R	R	S	S	R	R	S
	KI-113	2018/ 5/9	Skin	89	F	R	R	R	R	R	R	R	S	R	R	R	R	R	R	R	R	R	S	R	R	R	S	S	R	R	S
	KI-114	2018/ 5/10	Open pus	71	F	R	R	R	R	R	R	R	S	R	R	R	R	R	R	R	R	R	S	R	R	R	S	S	R	R	I
	KI-116	2018/ 6/1	Intratracheal sputum	74	M	R	R	R	R	R	R	R	S	R	R	R	R	R	R	R	R	R	S	R	R	S	S	S	R	R	I
	KI-120	2018/ 6/28	Intratracheal sputum	61	M	R	R	R	R	R	R	R	S	R	R	R	R	R	R	R	R	R	S	R	R	S	S	S	R	R	S
K	KI-164	2019/ 1/8	Sputum	85	M	R	R	R	R	R	R	R	S	R	R	R	R	R	R	R	R	R	S	S	R	R	S	S	R	R	R
	KI-188	2019/ 4/11	Sputum	84	M	R	R	R	R	R	R	R	S	R	R	R	R	R	R	R	R	R	S	R	R	R	S	S	R	R	R
	KI-189	2019/ 5/2	Wound	62	F	R	R	R	R	R	R	R	S	R	R	R	R	R	R	R	R	R	S	R	R	R	S	S	R	R	R
	KI-194	2019/ 5/17	Sputum	87	M	R	R	R	R	R	R	R	S	R	R	R	R	R	R	R	R	R	S	R	R	R	S	S	R	R	R
	KI-196	2019/ 6/6	Sputum	80	F	R	R	R	R	R	R	R	S	R	R	R	R	R	R	R	R	R	S	R	R	R	S	S	R	R	R
	KI-169	2019/ 2/4	Intratracheal sputum	80	M	R	R	R	R	R	R	R	S	R	R	R	R	R	R	R	R	R	S	S	R	S	S	S	R	R	R
	KI-176	2019/ 3/4	Open pus	88	F	R	R	R	R	R	R	R	S	R	R	R	R	R	R	R	R	R	S	S	R	S	S	S	R	R	I
L	KI-179	2019/ 3/7	Intratracheal sputum	90	M	R	R	R	R	R	R	R	S	R	R	R	R	R	R	R	R	R	S	S	R	S	S	S	R	R	R
	KI-191	2019/ 5/7	Feces	57	M	R	R	R	R	R	R	R	S	R	R	R	R	R	R	R	R	R	S	S	R	S	S	S	R	R	R
	KI-193	2019/ 5/13	Sputum	87	M	R	R	R	R	R	R	R	S	R	R	R	R	R	R	R	R	R	S	S	R	S	S	S	R	R	R

R (resistant to each antibacterial agent) was defined as follows; ampicillin (ABPC) (MIC >8 µg/mL), oxacillin (MPIPC) (MIC >2 µg/mL), azithromycin (AZM) (MIC >4 µg/mL), penicillin (PCG) (MIC >4 µg/mL), cefazolin (CEZ) (MIC >2 µg/mL), cefotiam (CTM) (MIC >4 µg/mL), erythromycin/clindamycin (EM/CLDM) (MIC >4.5 µg/mL), daptomycin (DAP) (MIC >0.5 µg/mL), cefdinir (CFDN) (MIC >1 µg/mL), cefepime (CFPM) (MIC >4 µg/mL), cefmetazole (CMZ) (MIC >8 µg/mL), flomoxef (FMOX) (MIC >4 µg/mL), imipenem (IPM) (MIC >2 µg/mL), meropenem (MEPM) (MIC >2 µg/mL), ampicillin/sulbactam (ABPC/SBT) (MIC >2 µg/mL), cefoperazone/sulbactam (CPZ/SBT) (MIC >8 µg/mL), piperacillin/tazobactam (PIPC/TAZ) (MIC >2 µg/mL), arbekacin (ABK) (MIC >1 µg/mL), gentamicin (GM) (MIC >2 µg/mL), clindamycin (CLDM) (MIC >2 µg/mL), minocyclin (MINO) (MIC >8 µg/mL), teicoplanin (TEIC) (MIC >1 µg/mL), vancomycin (VCM) (MIC >1 µg/mL), ciprofloxacin (CPFX) (MIC >2 µg/mL), levofloxacin (LVFX) (MIC >4 µg/mL) and fosfomycin (FOM) (MIC >128 µg/mL).

### Pulse-field gel electrophoresis (PFGE)

Pulse-field gel electrophoresis (PEGE) was carried out as described elsewhere [23–26]. A single colony of a test isolate was inoculated into 2 ml of Luria−Bertani broth and incubated with shaking at 37 °C for 16 h. The concentrations of the cell suspensions were adjusted with LB broth by using a spectrophotometer to an absorbance of 0.9 to 1.0 at 620 nm. Two-hundred microlitres of the adjusted cell suspension was centrifuged at 12000 ***g*** for 3 to 4 min, and the supernatant was removed. The pellet was resuspended in 300 µl of Tris-EDTA (TE) buffer (10 mM Tris HCl, 1 mM EDTA [pH 8]) and equilibrated in a 37 °C water bath for 10 min. To the cell suspension were added 4 µl of conventional (no. L-7386; Sigma-Aldrich, St. Louis, MO, USA) lysostaphin stock solution (1 mg ml^−1^ in 20 mM sodium acetate [pH 4.5]) and 300 µl of 1.5% (wt/vol) Certified Low-Melt agarose (Bio-Rad Laboratories, Hercules, CA, USA) in TE buffer (equilibrated to 60 °C), followed by gentle mixing. The mixture was dispensed into the wells of disposable plug moulds (Bio-Rad). The plugs were allowed to solidify at room temperature for 10 to 15 min. The plugs were removed and placed into a tube containing at least 3 ml of EC lysis buffer (6 mM Tris HCl, 1 M NaCl, 100 mM EDTA, 0.5% Brij-58, 0.2% sodium deoxycholate, 0.5% sodium lauroyl sarcosine) and incubated at 37 °C for at least 4 h. The EC lysis buffer was poured off, and 4 ml of TE buffer was added and incubated at 37 °C for 30 min. The TE washings were repeated at least three more times, and the plugs were stored at 4 °C.

A plug slice was cut to the desired comb size and equilibrated in 1×restriction buffer 30 °C for at least 4 h. After removal of the 1×restriction buffer, 2 µl of *Sma*I restriction enzyme (no. 1085A, 10 U µl^−1^; Takara Bio, Shiga, Japan) in 200 µl of 1×restriction buffer was added to each tube, and the tubes were incubated at 30 °C for 3 h. A PFGE 1% (wt/vol) agarose gel was prepared in 0.5×TBE from 10×Tris-borate-EDTA buffer (1M Tris-Base, 1 M boric acid, 0.02 M EDTA [pH 8.0]). The plug slices were loaded directly onto the end of the comb tooth before the comb was placed into the comb holder, and the equilibrated agarose was poured carefully into the gel casting platform. PFGE was performed using a contour-clamped homogeneous electric field apparatus, CHEF DR-II (Bio-Rad). The running parameters were as follows: 200 V (6 V cm^−1^); temperature, 14 °C; initial switch, 5 s; final switch, 40 s; time, 20 h. After the electrophoresis was completed, the gel was stained in a 0.3 µg ml^−1^ ethidium bromide solution for 1 h in a covered container.

Gels were photographed and digitized by the Printgraph AE-6914 (ATTO, Tokyo, Japan) and saved as a TIFF file for analysis with BioNumerics software (Applied Maths, Kortrijk, Belgium). The reference standard Lambda ladder (no. 1703635, Bio-Rad) was included in the first, seventh, and last lanes of each gel. Percent similarities were identified on a dendrogram derived from the unweighted pair group method using arithmetic averages and based on Dice coefficients. Band position tolerance and optimization were set at 1.25 and 0.5%, respectively [27]. A similarity coefficient of 80 % was selected to define the pulsed-field type (PFT) clusters after reviewing the epidemiologic data associated with each cluster of isolates.

### PCR amplification

Staphylococcal Cassette Chromosome *mec* (SCC*mec*) type [28] and coagulase gene type [29] were determined as described elsewhere. Isolates not identified as SCC*mec* types I–V were classified as nontypeable. Chromosome DNA was extracted by Cica Geneus DNA Extraction Reagent (Kanto Chemical, Tokyo, Japan). PCR to detect the Panton−Valentine leukocidin gene (*pvl*) was performed according to the method reported [30].

### Multilocus sequence typing (MLST)

Previously it was shown that MRSA strains from a major clonal group, as demonstrated by PFGE, have either the same sequence type (ST) or STs that are related to a single clonal complex (CC) [31–36]. In this study, CC (ST) was determined by selecting two representative strains from each group as classified into groups A to L by PFGE analysis. MLST was performed as described by Enright *et al*. [36]. The sequence type (ST) was determined by using the MLST database (https://pubmlst.org).

### Toxin production

For the detection of enterotoxin (SET- RPLA), toxic shock syndrome toxin-1 (TSST-1), and exfoliative toxin detection (EXT-RPLA), a commercial RPLA test kit was obtained from Denka Seiken (Tokyo, Japan). Culture supernatants were diluted serially two- and tenfold. Agglutination was observed after about 16 h of incubation.

### Usage status of antimicrobial agents

To understand the usage status of injectable antimicrobial agents (antibacterial agents), antimicrobial use density (AUD) and days of therapy (DOT) values were calculated. AUD and DOT were expressed as follows:

AUD (*DDDs/1000 patient-days)

= [Total dose (g) of antimicrobials used / DDD × Total days of hospital stay]×1000

DOT (DOTs/1000 patient-days)

= [Total days antimicrobials used / Total days of hospital stay]×1000

*The defined daily dose (DDD) was the value defined by the World Health Organization.

It is generally considered that the AUD/DOT rate will theoretically be 1.0 if antimicrobial agents are used properly [37].

### Statistical analysis

Comparisons of detection rate (%) of SCC*mec* types, CC (ST) types, and *coa* types were evaluated by Fisher’s exact test. Results with *P*<0.05 were assessed as statistically significant.

## Results and discussion

### Bacterial species with the highest detection rates at NHOKMC

**Fig. 1.**
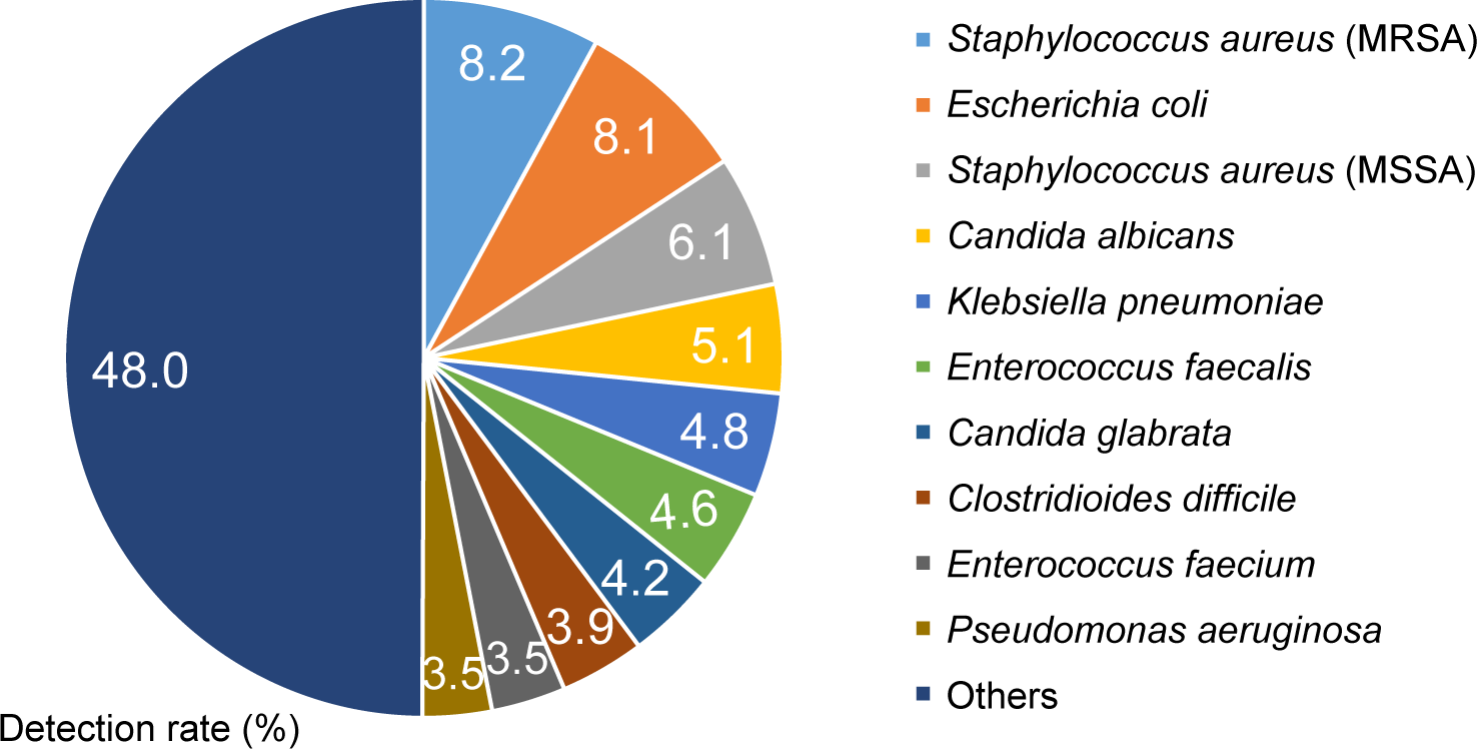
Detection rates of bacteria and fungi isolated at NHOKMC from 2017 through 2019. The total number of bacterial and fungal strain samples detected in NHOKMC in this period was 12932. The detection rates of the top ten species are shown.

The total number of bacterial and fungal strain samples detected in our hospital from 2017 through 2019 was 12932. The top ten species are shown in Fig. 1. These top ten species accounted for more than half (52.0%) of the samples. Among them, MRSA isolates accounted for the highest proportion (8.2% of the total number of specimens), followed by *Escherichia coli *, methicillin-sensitive *S. aureus* (MSSA), *Candida albicans*, and *Klebsiella pneumoniae *.

All of these isolates abound in daily life, and many are also resident as normal microbial flora in the human body. It is therefore suggested that ubiquitous microorganisms can be major pathogens of nosocomial infections, although it is a well-known fact. In several cases, MRSA strains were isolated from medical devices such as catheters (Table 1; see ‘origin of the sample’), suggesting that bacterial contamination of medical devices may not be eliminated even though various infection control methods have improved and medical devices have been handled carefully in recent years.

### PFGE profiles of 237 MRSA isolates from NHOKMC and their detection times

**Fig. 2.**
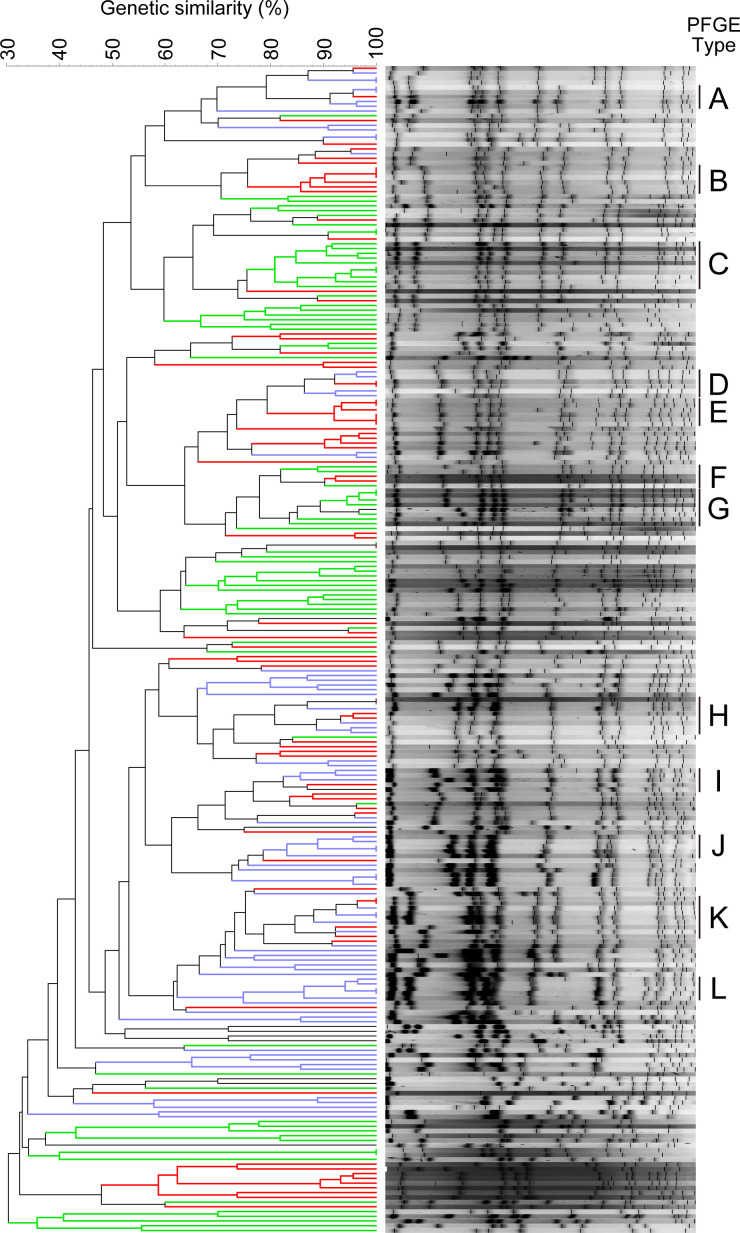
The PFGE profiles and phylogenetic tree of MRSA strains classified into A to L PFGE types. It was considered that there were 12 PFGE types (types A to L), each consisting of five or more MRSA clinical isolates (77 isolates) with over 80% genetic similarity, throughout 2017–2019. The colour coding of the phylogenetic tree indicates that green was isolated in 2017, red in 2018, and blue in 2019.

PFGE analyses were performed on 237 of the 1063 MRSA isolates isolated at our hospital from 2017 through 2019. These analyses revealed 12 PFGE types (types A to L), each consisting of five or more MRSA clinical isolates with over 80% genetic similarity throughout the 3 years. The PFGE profiles and phylogenetic tree are shown in Fig. 2. There were 77 MRSA isolates belonging to PFGE types A to L.

Considering the dates of isolation of the MRSA strains classified into their PFGE types A to L (‘Dates of sample collection’ in Table 1), it can be seen that the predominant PFGE type changes from year to year. That is, strains of PFGE types C, F, and G were isolated from January 2017 to the January 2018. Strains of types B and E were mainly isolated from July 2018 to the end of the same year, and those of types A, D, H, I, J, K, and L from the beginning of 2018 through all months of 2019. Thus, it seems that some specific PFGE types of MRSA strains may have been transmitted in the city or within the hospital because the same PFGE strain types predominated each year for a certain period. It is therefore inferred that MRSA strains derived from a specific origin and genetically related strains are carried among patients each year.

Table 1 also shows the susceptibility of 77 MRSA isolates of PFGE types A to L to various antibacterial agents. All isolates were sensitive to anti-MRSA drugs such as vancomycin (VCM), teicoplanin (TEIC), arbekacin (ABK), and daptomycin (DAP), indicating that these drugs are therapeutically effective against these isolates. These isolates were also sensitive to another anti-MRSA drug, linezolid (data not shown). Aside from these 77 isolates, all other isolated MRSA strains were sensitive to the above drugs (data not shown), suggesting that resistance to anti-MRSA drugs has not yet progressed in our locale. As a matter of course, none of the beta-lactam antibiotics showed antibacterial activity against any MRSA strains isolated. In addition, most isolates were resistant to quinolones (CPFX, LVFX), azithromycin (AZM), erythromycin (EM), and clindamycin (CLDM).

Since we classified five or more strains with over 80% genetic similarity as the same PFGE type in this study, there were some MRSA strains belonging to the same PFGE type which had different susceptibility to antibiotics. It is considered that the genetic evolution continues to progress in real time, although the MRSA strains of the same PFGE type are genetically closely related to each other. Thus, it is thought that recognizing the genetic typing of MRSA strains is important clue in understanding the genetic origin of the strain and its prevalence into the environment. However, it should also be necessary to carry out pharmacotherapeutic considerations when practicing infection prevention and treatment the infection because the sensitivities to the antibiotics do not completely match even if the strains are genetically related to each other.

### Molecular biological features of 237 MRSA isolates from NHOKMC

First, we examined the molecular biological findings of the 237 MRSA clinical isolates that we had subjected to PFGE analysis. We determined the SCC*mec* type, the coagulase gene type (*coa* type) and MLST (CC or ST). We also examined the presence of the *pvl* gene by the PCR method. The results are summarized in Tables 2–5.

**Table 2.** Transition of SCC*mec* types of the 237 MRSA isolates subjected to PFGE analysis

SCC*mec* Type	no. of isolates (rates, %)		
	2017	2018	2019
	(*n*=77)	(*n*=80)	(*n*=80)
I	0 (0)	0 (0)	4 (5)
II	30 (39)	26 (33)	31 (39)
III	0 (0)	0 (0)	1 (1)
IV	41 (53)	53 (66)*	38 (47)
V	5 (7)	0 (0)	4 (5)
nt	1 (1)	1 (1)	2 (3)

nt, nontypeable.

*P<0.05 vs 2019

**Table 3.** Transition of clonal types of the 237 MRSA isolates subjected to PFGE analysis

CC (ST)	No. of isolates (rates, %)		
	2017	2018	2019
	(*n*=77)	(*n*=80)	(*n*=80)
1 (1)	14 (18)	24 (30)	7 (9)**
5 (5, 764)	31 (41)	21 (26)	27 (34)
8 (8, 380)	21 (27)	22 (28)	27 (34)
30 (30)	1 (1)	0 (0)	0 (0)
121 (121)	4 (5)	0 (0)	1 (1)
nt	6 (8)	13 (16)	18 (22)

NT, nontypeable.

**P<0.01 vs 2018.

**Table 4.** Prevalence of *pvl* gene in 237 MRSA isolates subjected to PFGE analysis

*pvl*	No. of isolates (rates, %)		
	2017	2018	2019
	(*n*=77)	(*n*=80)	(*n*=80)
−	77 (100)	80 (100)	76 (95)
+	0 (0)	0 (0)	4 (5)

**Table 5.** Transition of *coa* genotypes of the 237 MRSA isolates subjected to PFGE analysis

*coa* Type	No. of isolates (rates, %)		
	2017	2018	2019
	(*n*=77)	(*n*=80)	(*n*=80)
1	2 (3)	0 (0)	0 (0)
2	11 (14)	27 (34)**	33 (41)**
3	23 (30)	29 (36)	32 (40)
4	2 (2)	1 (1)	4 (5)
5	20 (26)	0 (0)**	2 (3)**
7	11 (14)	20 (25)	8 (10)*
8	2 (3)	0 (0)	0 (0)
nt	6 (8)	3 (4)	1 (1)

NT, nontypeable.

*P<0.05 vs 2018.

**P<0.01 vs 2017.

Examination of the SCC*mec* genotypes of the clinically isolated MRSA strains in NHOKMC during the 3 years from 2017 through 2019 revealed that 92 % of the strains belonged to SCC*mec* type II or type IV throughout the 3 years (Table 2). As far as the SCC*mec* genotype is concerned, therefore, it is considered that MRSA of a specific genotype is widely distributed in our locale. For unknown reasons, more MRSA strains having SCC*mec* type IV were isolated in 2018 than in the other years.

Another study of MRSA isolated from patients with impetigo in Kagawa Prefecture, Japan, was reported [38]. In that study, a survey showed that MRSA isolates having SCC*mec* type II or IV were main isolates, but more MRSA isolates having SCC*mec* type V were also isolated comparing with the results of our study. Thus, the distribution situation of MRSA seemed to be slightly different from that in our area.

As a result of our MLST analysis (Table 3), CC1, CC5, and CC8 clones were predominantly isolated throughout the 3 years. In 2019, the prevalence of CC1 clones was found to be significantly reduced. In fact, the proportion of patients who developed infectious diseases with MRSA isolated as the main pathogen was most frequently caused by MRSA with a clonal complex of CC5 and CC8 in our hospital during 2017 through 2019 (data not shown). We therefore think it necessary to keep an eye on the trends of MRSA strains with a CC5 or CC8 clonal complex as pathogens that cause infectious diseases directly.

Moreover, the MRSA strain USA300 is known to be highly pathogenic and to pose a public health problem in the United States [39–41]. This strain belongs to CC8 (ST8) and is characterized by the ability to produce the PVL virulence factor. We then examined 237 MRSA strains by PFGE for the presence of the *pvl* gene. As shown in Table 4, none of the 157 MRSA strains isolated from 2017 through 2018 carried the *pvl* gene, but four of the 80 MRSA strains isolated in 2019 carried it. Of those four strains, three were CC8 (ST8) MRSA strains, suggesting that they may be the same clonal strain as USA300. From this result, we cannot rule out the possibility that a new pathogenic MRSA strain has invaded our local area. We therefore think that it is necessary to continue to carefully monitor it in the future.

Our examination of the *coa* type revealed that nearly 90% of the MRSA isolates carried the type 2, 3, 5, or seven *coa* gene in each year (Table 5). Among these, from 2018 through 2019, the *coa* types 5 and 7 MRSA isolates seem to have significantly declined. MRSA strains with a specific *coa* gene seem to be widely distributed in our locale.

We further investigated whether MRSA strains isolated from another hospital (Saiseikai Kure Hospital), which is in the same area as our hospital, had similar molecular biological characteristics. Although the data were from only 25 MRSA strains isolated in 2017, all of the MRSA isolates had either a type II (68%) or type IV (32%) SCC*mec* genotype, and their PFGE types were close to PFGE type C shown in Fig. 2. Of these 25 MRSA isolates, 64% had a CC5 clonal complex (12% CC1; 8% CC8; 16% nontypeable) and 68% of MRSA isolates had the type 2 *coa* gene (20% type 3; 12% type 7). Considering these data together, it is likely that MRSA strains with specific molecular biological characteristics may be predominant in our local area.

### Toxin production of 77 MRSA isolates classified into A to L PFGE types

Next, we investigated the bacteriological features, including the production of various staphylococcal toxins, of 77 MRSA isolates classified into A to L types by PFGE analysis. The results are summarized in Table 6. Molecular biological findings are also included in this table.

**Table 6.** Molecular biological and bacteriological features of 77 MRSA isolates analysed by PFGE

Year	No. of isolates	PFGE type	Major genotypes included in each PFGE type				No. of isolates producing each virulence factor belonging to each PFGE type (ratio, %)						
			SCC*mec*	CC	*coa* type	*pvl*	Enterotoxin A	Enterotoxin B	Enterotoxin C	Enterotoxin D	TSST-1	Exfoliative toxin A	Exfoliative toxin B
2017	10	C	2	5	2	0 (0)	0 (0)	6 (60)	0 (0)	0 (0)	0 (0)	0 (0)	0 (0)
2017	7	G	4	1	7	0 (0)	3 (43)	1 (14)	0 (0)	0 (0)	0 (0)	0 (0)	0 (0)
2017–2018	5	F	4	1	7	0 (0)	3 (60)	0 (0)	1 (20)	0 (0)	1 (20)	0 (0)	0 (0)
2018	6	B	2	5	2	0 (0)	0 (0)	6 (100)	2 (33)	0 (0)	0 (0)	0 (0)	0 (0)
2018	6	E	4	1	7	0 (0)	5 (83)	0 (0)	0 (0)	0 (0)	0 (0)	0 (0)	0 (0)
2018–2019	5	A	2	5	2	0 (0)	0 (0)	4 (80)	1 (20)	0 (0)	0 (0)	0 (0)	0 (0)
2018–2019	6	D	4	1	7	0 (0)	4 (67)	0 (0)	0 (0)	0 (0)	0 (0)	0 (0)	0 (0)
2018–2019	8	H	4	8	3	0 (0)	0 (0)	1 (13)	2 (25)	0 (0)	2 (25)	0 (0)	0 (0)
2018–2019	5	I	4	8	3	0 (0)	0 (0)	0 (0)	0 (0)	0 (0)	0 (0)	0 (0)	0 (0)
2018–2019	9	K	2	5	2	0 (0)	0 (0)	8 (89)	4 (44)	0 (0)	1 (11)	0 (0)	0 (0)
2019	5	J	4	8	3	0 (0)	0 (0)	0 (0)	2 (40)	0 (0)	2 (40)	0 (0)	0 (0)
2019	5	L	2	5	2	0 (0)	0 (0)	0 (0)	5 (100)	0 (0)	5 (100)	0 (0)	0 (0)

As mentioned in the section showing the results of PFGE analysis, it can be seen that the PFGE types of the MRSA strains isolated from 2017 through 2019 change from year to year. Almost all MRSA isolates carried SCC*mec* type II or type IV. Our examination of the toxin production of each MRSA isolate revealed that none of the 77 isolates produced exfoliative toxins A or B in this study. Only a few isolates produced toxic shock syndrome toxin-1 (TSST-1), which causes cytokine storm-like toxic shock syndrome [42], but all of the five MRSA isolates belonging to PFGE type L isolated in 2019 produced TSST-1. These isolates also produced staphylococcal enterotoxin C. As can be seen from the PFGE profile shown in Fig. 2, the migration patterns of the gene fragments of the five MRSA isolates belonging to PFGE type L were very similar to each other. In addition, the antibacterial sensitivity profile of these strains shown in Table 1 were also almost the same. Thus, it is considered that these strains (PFGE type L) may be originated from the same clone. These results may indicate that new MRSA pathogenic strains have been emerging in our living area in recent years. We should therefore pay attention to the trends of these MRSA strains in the future.

On the other hand, the production levels of various staphylococcal enterotoxins were observed in each PFGE type except for PFGE type I at a certain rate. Interestingly, there seemed to be a correlation between the production of some staphylococcal enterotoxins and the molecular biological characteristics of the MRSA strains isolated in this study.

First, we found that most MRSA isolates carrying SCC*mec* genotype II produced enterotoxin B and/or C and that most isolates carrying SCC*mec* genotype IV produced enterotoxin A. Next, all the isolates in which the production of staphylococcal enterotoxin A (SEA) was detected had CC1 in MLST. In addition, 22 of 26 MRSA isolates (about 85%) in which staphylococcal enterotoxin B (SEB) production was detected carried the type 2 *coa* gene. Moreover, among the 17 isolates in which staphylococcal enterotoxin C (SEC) production was observed, 12 isolates had CC5 and five had CC8 in MLST.

Varshney *et al*. reported the frequencies of various staphylococcal toxin genes among different clonal backgrounds [43]. That report showed that the frequency of detection of the SEC gene was relatively high in MRSA strains having CC5 and CC8 in MLST. This result seemed to correlate with the CC type of the MRSA isolates that showed SEC productivity in our study. However, it was reported that the SEA gene was not detected in the MRSA strain having CC1 in MLST. This finding is different from the relationship between staphylococcal enterotoxin productivity and MLST obtained in our study.

Similar studies were reported by Rasmussen *et al*. [44]. Considering the findings of those studies together, it cannot be said unconditionally that only MRSA strains carrying a specific MLST produce a specific type of staphylococcal enterotoxin. Thus, molecular biological properties such as MLST indicate only the genetic background between MRSA strains, and it seems that we may not be able to evaluate virulence based on these characteristics alone. However, we think that combining various properties such as toxin production and genotype may lead to a more accurate understanding of MRSA characteristics. We also believe that a detailed understanding of MRSA characteristics may lead to support for actions in the medical field such as those leading to the selection of more appropriate therapeutic agents.

### Relationship between antimicrobial usage and changes in MRSA isolation rates

Finally, we would like to mention the relationship between antimicrobial usage over time and changes in MRSA isolation rates. Appropriate use of antibacterial agents is a matter that medical staff should always keep in mind in order to inhibit the spread of both bacteria that cause infectious diseases and drug resistance. In Japan, the National Action Plan on Antimicrobial Resistance (AMR), established by the Japanese government on 5 April 2016, also strongly demands the optimization of the use of antibacterial agents and tries to stop the resistance of causative bacteria against antibacterial agents. In addition, the appropriate use of antibacterial agents improves the quality of medical care, improves the effectiveness and safety of antibacterial agents, and reduces medical expenses, which is beneficial in terms of medical economics.

**Fig. 3.**
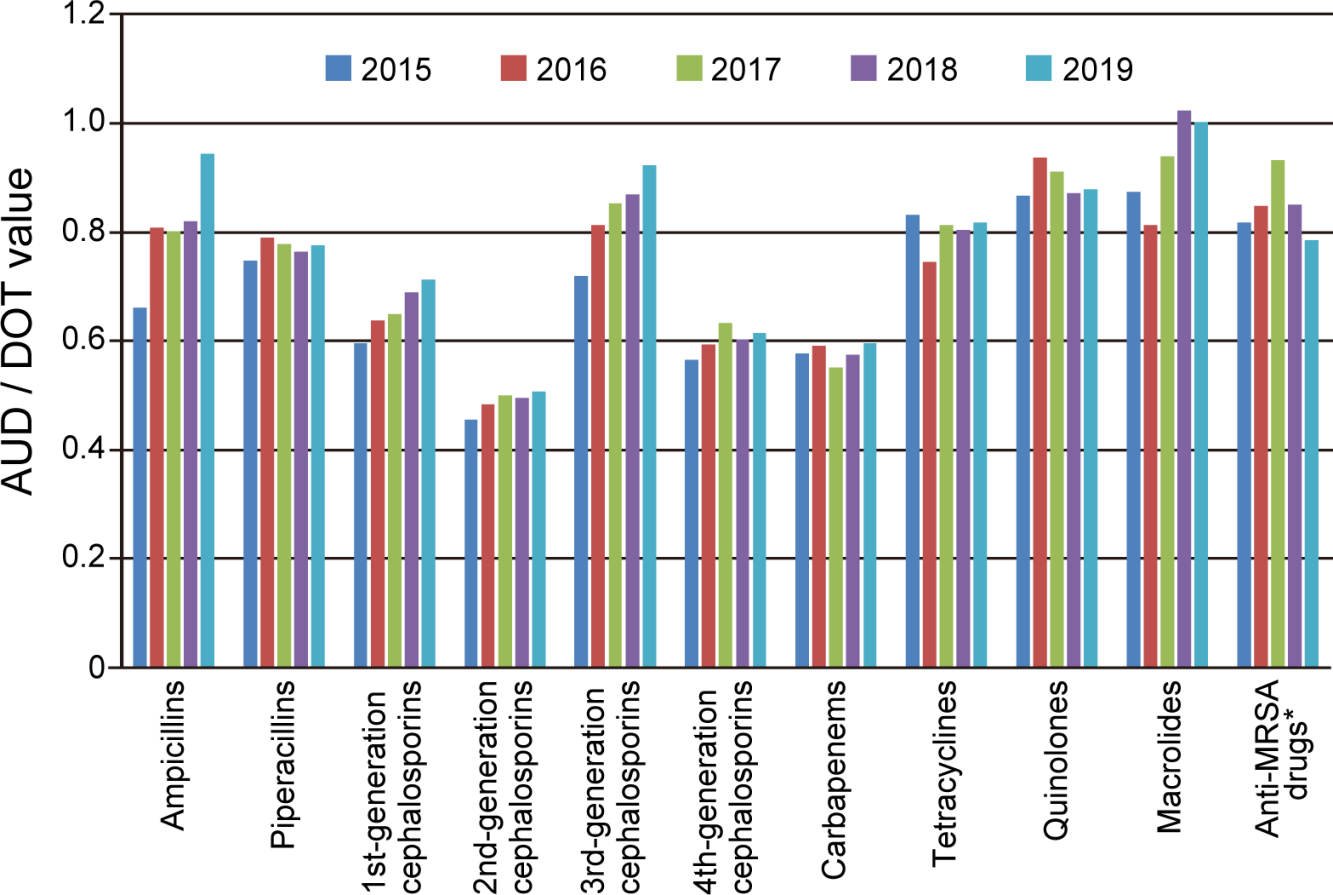
Annual changes in AUD/DOT values against various kinds of antibacterial agents used at NHOKMC. AUD/DOT values were calculated for various kinds of antibacterial agents used between 2015 and 2019 at NHOKMC. AUD/DOT values were calculated according to the method described in the text. *Anti-MRSA drugs included vancomycin, teicoplanin, daptomycin, and linezolid.

In our hospital, we evaluate the appropriateness of the use of various antibacterial agents every year by using AUD/DOT values as an index. Information must be shared among the clinical staff of the infection control team as necessary to further optimize AUD/DOT value. Fig. 3 shows the changes in the AUD/DOT values of various kinds of antibacterial agents during the 5 years from 2015 to 2019. Due to the efforts of medical staff, the AUD/DOT value has increased in the use of almost all antibacterial agents, and the use of some agents has approached the ideal value of 1.0. Thus, we believe that the use of antibacterial agents is being optimized year by year.

**Fig. 4.**
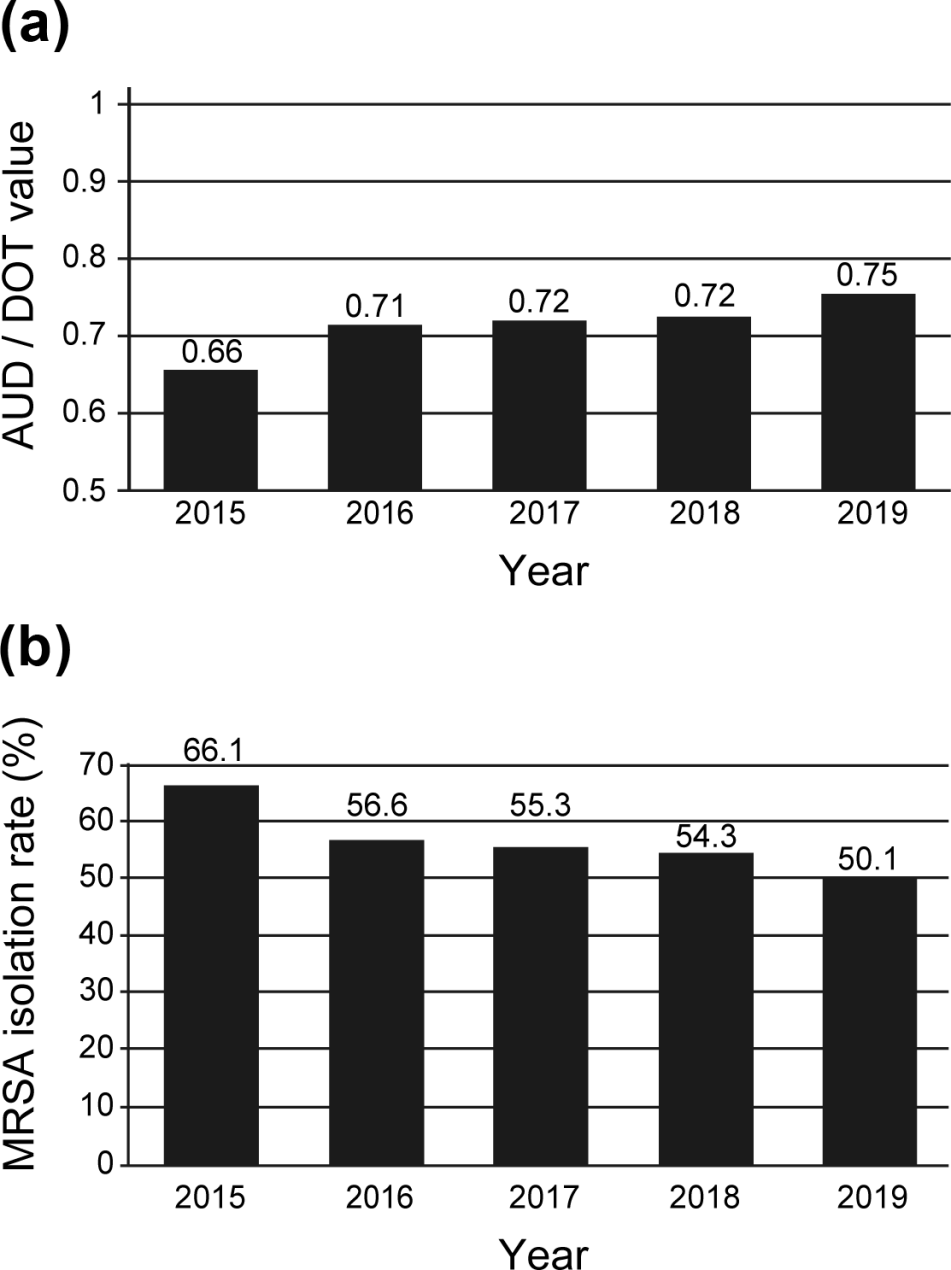
Annual changes in total AUD/DOT values and in the MRSA isolation rate. (a) Total AUD/DOT values were calculated for all antibacterial agents used between 2015 and 2019 in NHOKMC. (b) Annual changes in the MRSA isolation rate during 2015 through 2019 at NHOKMC. The MRSA isolation rate (%) was calculated as the rate of the number of MRSA isolates to the total number of *S. aureus* isolated from the patients at our hospital.

We comprehensively investigated how the isolation rate (%) of MRSA changed with these changes in the situation at NHOKMC. We define the proportion of MRSA strains among the *S. aureus* clinical strains isolated in NHOKMC as MRSA isolation rate (%). The results are shown in Fig. 4. Our calculation of the total AUD/DOT value for the use of all antibacterial agents showed that use has certainly increased from year to year (Fig. 4a). According to the increase in the total AUD/DOT value, we found that the MRSA isolation rate (%) in our hospital decreased year to year (Fig. 4b). These results are considered to be important data that clearly show that the isolation rates of pathogens can be reduced by optimizing the use of antibacterial agents. In addition, Nakamura *et al.* reported that closer communication and better feedback between antimicrobial stewardship teams and prescribers lead to the optimal use of antibacterial agents [37]. In this sense, we believe that the current efforts of NHOKMC medical staff need to continue to be strengthened.

In the future, we would like to collect more data and report on how the MRSA isolation rate has changed. We would like also to proceed with similar clinical research on major pathogens other than MRSA.
